# The importance of neighborhood ecological assets in community dwelling old people aging outcomes: A study in Northern Portugal

**DOI:** 10.3389/fnagi.2015.00156

**Published:** 2015-09-01

**Authors:** Alice M. Bastos, Carla G. Faria, Emília Moreira, Diana Morais, José M. Melo-de-Carvalho, M. Constança Paul

**Affiliations:** ^1^Instituto Politécnico de Viana do Castelo – Escola Superior de Educação, Unidade de Investigação e Formação sobre Adultos e Idosos, Viana do CasteloPortugal; ^2^Instituto de Ciências Biomédicas Abel Salazar, Unidade de Investigação e Formação sobre Adultos e Idosos, Universidade do PortoPorto, Portugal

**Keywords:** ecological assets, activities of daily living, cognitive functioning, depression, aging outcomes

## Abstract

Human development is a bidirectional, person-context relational process, but scarce evidence is available about the relation between the individual variability across the life-span and the neighborhood ecological assets. Therefore, it is important that research focus not only on personal characteristics but on ecological assets as well. This way this study aims to analyze the association between neighborhood ecological assets categorized into four dimensions: human, physical or institutional, social or collective activity, accessibility, and the individual functioning. A 3% sample of residents aged 65 years and older in two downtown and three uptown parishes stratified by age and sex was interviewed at home using a protocol that included the Portuguese version of the Barthel Index in basic activities of daily living (BADL), the Lawton Instrumental Activities of Daily Living Scale (IADL), the Mini Mental State Examination (MMSE), and the Geriatric Depression Scale-15 items (GDS) for evaluating functionality, cognitive performance, and depression. The 162 participants were aged on average 75 years (sd = 7.0), 54% were women and 90% had less than 7 years of education. The majority of participants were independent in BADL (*M* = 90; sd = 17.7) and moderately dependent in IADL (*M* = 13, sd = 6.0), 20% showed cognitive impairment and a mean score of 8 (sd = 2.1) in GDS-15. After controlling for the effect of socio-demographic characteristics, functionality, and cognitive performance decreases in persons with worst outdoor mobility. On the other hand depressive symptoms are less common as the number of recreation opportunities, namely associative groups (cultural, educative, professional), increases. These results suggest that aging policies and practices must be ecologically embedded.

## Introduction

Currently, human aging is seen as one of the highest challenges of the XXI century. In the western world, we won about 33 years in average life expectancy, which rose from about 46 years, in 1900, to about 79 years in the end of the century (Walker, 2010; Hooyman and Kiyak, 2011). However, longevity advances are not being accompanied by a similar transformation in societal terms, leading Baltes and Smith (2003) to question our capacity to conciliate longevity and dignity, especially in fourth age. Furthermore, considering the theoretical and methodological knowledge advance, in the Social and Behavioral Sciences, in terms of the *life course perspective, aging is a lifelong process*, situated in an historical space and time (Elder, 1974; Elder and Shanahan, 1998). Also, according to the *life-span developmental psychology* (Baltes, 1987, 1997; Baltes et al., 2006), development is not completed at adulthood (maturity); rather ontogenesis extends across life span. Thus, aging is a dynamic process of gains and losses, which follows from the biological and cultural architecture.

In the present study, we focused on the following aging outcomes: functional dependency, cognitive functioning, and depression. These outcomes have mostly been analyzed under a socio-demographic characteristics perspective. Functional dependency has been tightly associated to age, gender, education, and marital status. This way, dependent old people are mainly women, widows, people living alone and with low education (Murtagh and Hubert, 2004; Formiga et al., 2007; Santos et al., 2007; Espigares et al., 2008; Torres et al., 2009; Espigares and Torres, 2010; Espelt et al., 2010; Nunes et al., 2010). Functional dependency prevalence takes different shapes, as it refers to basic activities of daily living (BADL) or to instrumental activities of daily living (IADL). The majority of old people is independent in BADL (Costa et al., 2006; Schneider et al., 2008; Nakatani et al., 2009; Espigares and Torres, 2010) but those who are 70 or older are more prone to be dependent in IADL, and if they are older than 80 years, they might be dependent on both IADL and BADL (Nunes et al., 2010). Research has shown that both the prevalence and the rate of dependency increase with age (Espigares et al., 2008; Espelt et al., 2010) and that dependency is associated with several factors as cognitive impairment, depression, neurological diseases, back, neck and shoulders pain, osteoarthritis, chronic back problems, osteoporosis, more medication, lower economic resources (Murtagh and Hubert, 2004; Formiga et al., 2007; Santos et al., 2007; Schneider et al., 2008; Nunes et al., 2010). On the other hand age is not the most important factor for cognitive impairment measured by the Mini Mental State Examination (MMSE; Green et al., 2008; Karlamangla et al., 2009), but it has been found a global decline in cognitive functioning with age (Hooren et al., 2007; Bourne et al., 2010; Martínez-Vidal et al., 2011). Beyond age, other factors have been associated to cognitive decline in old people, as gender, marital status, education, social support network, socioeconomic status, and lifestyle (Hooren et al., 2007; Karlamangla et al., 2009). In general, a worse cognitive functioning is present in men, old people living alone or institutionalized, with a small social network confined to family, with little physical activity and having scarce socioeconomic and educational resources (Hooren et al., 2007; Green et al., 2008; Lindwall et al., 2008; Karlamangla et al., 2009; Oliveira et al., 2011). Although it is consensual that age brings a decrease in cognitive functioning, cognitive resources that are built in childhood and adulthood can prevent it or delay it (Yount, 2008). The other aging outcome considered in this study is depression, considered the most prevalent psychopathological disorder in old age (Seby et al., 2011). Depression has been related to several sociodemographic variables, such as age, gender, marital status, socioeconomic status, and education. Higher levels of depression occur mostly in old people aged 70 or older (Beekman et al., 2002; Chou and Chi, 2005; García-Peña et al., 2008), in old women (Copeland et al., 2004; McCusker et al., 2005; Tsai et al., 2005; García-Peña et al., 2008; Weele et al., 2009; Oliveira et al., 2011; Seby et al., 2011), old people living alone (Tsai et al., 2005; Oliveira et al., 2011; Seby et al., 2011), and old people with lower education and low socioeconomic resources (Tsai et al., 2005; Perrino et al., 2009; Akyol et al., 2010; Engmann, 2011). Besides the sociodemographic profile, other risk factors have been identified, such as previous history of depression, lack of social support perception, narrow social network, one or more physical diseases, adverse life events, poor quality of life perception, and lack of a regular spiritual practice (Copeland et al., 2004; Chou and Chi, 2005; Koizumi et al., 2005; McCusker et al., 2005; Tsai et al., 2005; Wilson et al., 2007; García-Peña et al., 2008; Akyol et al., 2010; Seby et al., 2011). The three analyzed dimensions in the present study – functionality, cognitive functioning, and depression – have a central role on aging, since they interact and influence each other, acting as roots for the successful or unsuccessful aging.

In a *developmental systems perspective*, Lerner (2002, 2012) has developed a series of studies about the positive development of young adults, highlighting the role of the “ecological assets,” wherein “assets can be conceived of within individuals, in the physical space, and emerging in the dynamic between the two” (Theokas and Lerner, 2006, p. 62). Regarding the Study of Positive Youth Development, these authors (2006) proposed four classes of observed ecological assets to organize the actual resources and opportunities in the environments – human, physical or institutional, collective activity, and accessibility. The concept definition, according to the above mentioned authors are as follows: (1) *Human resources* are defined as the strengths, skills, talents, and facilities of people and as instantiated by the roles they have. The characteristics, activities, and behaviors of individuals provide a manifestation of the social norms of a particular context; (2) *Physical or institutional resources* are intended to document opportunities for learning, recreation, and engagement with individuals and the physical world around oneself and, as well, for providing routines and structure for individuals. At the *neighborhood level*, the presence of libraries, community centers, and cultural experiences that are within walking distance, or available through public transportation may increase their use and thus their potential benefits; (3) *Collective activity* is intended to document mutual engagement between community members, parents, youth, schools personnel, and institutions of society. These organizations, groups, or mutual activities represent the combined efforts and actions of different sets of individuals, documenting ties and networks of a community’s associational life and the climate of the key context of development; (4) *Accessibility* intents to document the ability of residents to partake of human resources and resource opportunities in the context. Accessibility can be conceptualized and operationalized in multiple ways: (a) documents physical ease of access and can refer to the transportation capacity and hours of operation of local businesses, infrastructure, or cultural institutions in a local community; (b) can refer to the potential of youth to interact with the adults in the setting or what is the ratio of adults to children in a given neighborhood or how long has a family lived in a neighborhood; (c) can refer also to the safety of the physical environment and free of dangers.

Given these results, what happens in the studies about aging? Aging is a process that implies a series of alterations in the biological, psychological, and social domains. The multiple profiles that can result from the several combinations occurring among these alterations make aging a multifaceted process, shaped by previous development and, in which the individual has a proactive role, in that, in the interaction with the environment, he can become his own aging’s author. This way, different life styles in different environments result in different aging processes, which approach or distance themselves from active or successful aging. Researches on Environmental Gerontology (Wahl et al., 2012) made a first theoretical approach to the works of Lawton and the lifespan developmental perspective in a conceptual article that aims to guide future research agenda and propose theoretical propositions to an integrative model of Aging Well. Nonetheless, these theoretical propositions were only discussed in theoretical terms and not empirically tested. According to Dannefer (2003, 2009), over the course of life, people accumulate advantages or disadvantages that may optimize or limit the aging process.

In this context, we may ask: can the life-span developmental perspective using the methodology adopted by Theokas and Lerner (2006) contribute to a better understanding of aging in place? The aim of this study is to demonstrate that ecological assets are associated with aging outcomes, namely functionality, cognitive functioning, and depression, in an urban environment.

## Materials and Methods

### Participants

Sample size was estimated to be 3% of the resident population aged 65 years and older living in the five parishes/inner city of Viana do Castelo based on estimates of inter-census population for 2006, stratified by age and sex. The IPVC scientific committee approved the study and all persons willing to participate gave informed consent. Persons were interviewed at home by a trained researcher and a stakeholder (usually the priest or the president of the parish committee) in each parish indicated the first person to be interviewed. This person in turn pointed × persons, usually ranging from 4 to 10, and from them we randomly selected 50% to initiate the process. This can be viewed as cluster sampling with clusters with variable size.

### Data Collection

The collected information included socio-demographic characteristics, person’s daily functioning and household and neighborhood relationships. The Lawton instrumental activities of daily living scale (IADL-Lawton) was used to assess functionality, the MMSE for cognitive performance and the Geriatric Depression Scale (GDS) for depression. Ecological assets by parish included physical facilities (Elder and health facilities, nursing homes and culture/sports/recreation facilities among others) and social resources (Sports/cultural, health support associations, educative associations, and local/professional groups) based on a social diagnostic report of the City Council/Interparish committee (Relatório Social, 2008). Information focusing the resources and social necessities of each selected parish was analyzed. Accessibility indicators were based on the inter-census population estimates: dependency index (persons aged 65 years or more/persons aged 15–64 years), longevity index (proportion of persons 85 years or more among those aged at least 65 years), and characteristics of the public transport network based.

### Data Analysis

After data description, involving personal, micro, and macro-environmental characteristics, downtown and uptown residents were compared using non-parametric tests, the chi-square for categorical variables and the Kruskal–Wallis for ordinal/discrete variables. Hierarchical regression models were used to analyze predictors of functionality (IADL-Lawton), cognition (MMSE), and depression (GDS). The first block included socio-demographic characteristics; a second block included household and neighborhood assets that were entered in the model using a stepwise procedure; in the same way a third and fourth blocks included ecological assets, namely physical and social community resources and accessibility. Since the distribution of the MMSE scores did not follow the normal distribution we used a transformed variable – the square root of the number of errors [√(30 – score in MMSE = number of errors; Jacquin-Gada et al., 1997)].

## Results

A total of 162 persons were interviewed in the five parishes (*n* = 85 in downtown and *n* = 77 in uptown) and half of them are aged between 65 and 74 years old (**Table 1**). Most of them are women (54.3%). About 9.3% are illiterate, most are married (46.9%) or widower (40.1%). Overall 56.8% had blue collar occupations during their professional life, being this proportion higher among the uptown residents comparing to downtown (*p* = 0.018). Participants lived in the same parish for 63 years (sd = 31.5) and the majority considered to have good household conditions and a good relationship with neighbors (80.9%), being this proportion even higher among the downtown residents (*p* = 0.035). Overall 28.4% of participants report difficulties in outdoor mobility.

**Table 1:** Human resources and microenvironment accessibility resources.

Characteristics	Downtown (*n* = 85)		Uptown (*n* = 77)		All (*n* = 162)		Test *(p)^†^*
	*n*	%	*n*	%	*n*	%	
**Socio-demographic characteristics**				
Age *M* (sd)	74.5	(7.0)	74.6	(7.1)	74.6	(7.0)	
65–74	48	56.5	44	57.1	92	56.8	0.3 (0.9)
75–84	29	34.1	24	31.2	53	32.7	
85+	8	9.4	9	11.7	17	10.5	
Gender (% of women)	40	47.1	48	62.3	88	54.3	3.8 (0.051)
Education *M* (sd)	5.0	(3.4)	2.9	(1.4)	4.0	(3.0)	
0	5	5.9	10	13.0	15	9.3	23.4 (<0.001)
1–3	14	16.5	27	35.1	41	25.3	
4–6	49	57.6	40	51.9	40	54.9	
7+	17	57.6	0	0.0	17	10.5	
Marital status							
Married	41	48.2	35	45.5	76	46.9	
Widower	32	37.6	33	42.9	65	40.1	
Single/separated/divorced	12	14.1	9	11.7	21	13.0	0.5 (0.8)
Type of occupation							
White collar	28	32.9	11	14.3	39	24.1	8.0 (0.018)
Blue collar	44	51.8	48	62.3	92	56.8	
Household	13	15.3	18	23.4	31	19.1	
**Household and Neighborhood**				
Neighborhood stability* *M* (sd)	61.5	29.5	64.6	33.8	62.9	31.5	0.5 (0.5)
Good household conditions	75	88.2	61	79.2	136	84.0	2.4 (0.1)
Good relationship with neighbors	74	87.1	57	74.0	131	80.9	4.4 (0.035)
Difficulty in outdoor mobility	19	22.4	27	35.1	46	28.4	3.2 (0.1)
**Outcomes**				
Functionality in IADL (Lawton)	12.9	(5.6)	13.5	(6.0)	13.1	(6.0)	
Cognitive performance [Mini Mental State Examination (MMSE)]	26.5	(4.0)	24.9	(4.7)	25.7	(4.4)	
Depression [Geriatric Depression Scale (GDS)]	7.6	(2.0)	8.6	(2.0)	8.1	(2.1)	

*Lifetime lived in the neighborhood (%); ^†^X^2^ for categorical variables and Kruskal–Wallis for continuous variables.

The MMSE scores were associated with age (*p* < 0.001) and there were significant differences between the youngest and oldest groups; the IADL-Lawton score increased with age and the eldest had a higher score than the remainder. The GDS score were associated with age (*p* < 0.05) and there were significant differences between the two younger groups (65–74 vs. 75–84).

**Table 2** shows the physical, social, and accessibility resources by each parish in downtown and uptown. Downtown parishes have more physical resources for childhood/ adolescence, for education and more culture/sports and recreation facilities, as well as more sports and cultural groups and local/professional groups.

**Table 2:** Macroenvironmental physical, social, and accessibility resources by parish.

	Downtown		Uptown		
	P1	P2	P3	P4	P5
**Physical resources**			
Elder facilities	1	5	1	0	2
Health facilities	2	5	0	1	2
Resources for childhood/adolescence	1	10	2	1	3
Resources for education	6	8	2	4	6
Culture/sports/recreation facilities	4	5	0	0	0
**Social resources**			
Sports/cultural groups	21	39	7	5	13
Health support associations/groups	0	3	0	0	0
Educative associations/groups	0	1	0	0	0
Local/professional groups	6	8	0	0	2
**Accessibility**			
Population (%)	32 (19.8)	53 (32.7)	21 (13.0)	26 (16.0)	30 (18.5)
Public transportation (Bus)					
Number	1.8	2.75	1	1	1.25
Minimum frequency (minutes)	30	10	30	10	15
Dependency index	0.28	0.21	0.23	0.16	0.16
Longevity index (%)	8.9	10.4	8.4	6.6	9.8

*Physical resources: elders facilities: nursing homes; day care; home care; health facilities: hospitals; health centers; resources for childhood/adolescence: home care centers, day care centers; resources for education: kindergartens; schools; culture/sports/recreational opportunities: library; museums; recreational centers; civic centers; associations of retired persons.*

*Public transportation number: 1 = 1 Bus line for 12 h; dependency index: persons aged 65 or more years/persons aged 15–64 years; longevity index: persons aged 85 or more years/persons aged 65 or more years.*

Concerning accessibility, downtown parishes have a higher number of Bus Transportation in their territory. Minimum frequency of bus lines is lower in a downtown and in an uptown parish. Both dependency and longevity indexes are higher in downtown parishes.

The hierarchical regression models of functionality (IADL-Lawton), cognitive performance (MMSE) and depression (GDS) including a first set of personal characteristics and a second set of ecological assets (stepwise inclusion) indicated that 37% of the variability in functionality on the IADL-Lawton is explained by socio-demographic characteristics (age and marital state), household/neighborhood characteristics (outdoor mobility) and accessibility (dependency index and longevity index; **Table 3**).

**Table 3:** Multiple regression models* for functionality, cognitive performance, and depression including micro and macro environmental resources.

Characteristics	Functionality	Cognition	Depression
Socio-demographic characteristics			
Age (years)	0.20^a^	0.04^b^	0.03
Male vs. Female	0.94	-0.25	-0.28
Education (years)	0.76	-0.07^c^	0.01
Married vs. others	-2.45^b^	-0.27	-0.95^c^
White collar vs. others	-0.30	-0.33	-0.67
** *R*^2^**	**0.15**	**0.26**	**0.14**
Household and neighborhood			
Outdoor mobility (difficult vs. others)	4.73^a^	0.42^c^	
Household conditions (good vs. others)			
Neighbors relation (good vs. others)			
Neighborhood stability**			
** *R*^2^**	**0.32**	**0.30**	
Physical and social resources			
Physical			
Social			-0.02^c^
** *R*^2^**			**0.18**
Accessibility			
Public transport net (number)			
Dependency index	-25.1^c^	-4.50^c^	
Longevity index	0.77^b^		
** *R*^2^**	**0.37**	**0.32**	

*Setwise regression models: first block – human resources (all variables enter the model); variables in the next three blocks entered the model using a stepwise procedure; *R*^2^: coefficient of determination; **Lifetime lived in the same house (%); ^a^*p* < 0.001, ^b^*p* < 0.01, ^c^*p* < 0.05; physical and social resources: sum of values in **Table 2**.

Having difficulties in outdoor mobility (*p* < 0.001) and living in a parish with a lower dependency index (*p* < 0.05) and a higher longevity index (*p* < 0.01) is related to a higher score in IADL-Lawton (indicating worse levels in functionality), after controlling for the effect of socio-demographic characteristics.

Overall 32% of the variance in the MMSE (number of errors) is explained by the inclusion of socio-demographic characteristics (age and education), household and neighborhood characteristics (outdoor mobility) and accessibility (dependency index). Having difficulties in outdoor mobility (*p* < 0.05) and living in a parish with a lower dependency index (*p* < 0.05) is related to a higher number or errors in MMSE, after controlling for the effect of socio-demographic characteristics.

A total of 18% of the variance in the GDS is explained by inclusion of socio-demographic characteristics (marital state) and social resources. After controlling for demographic characteristics, depression decreases with the number of social resources (*p* < 0.05).

## Discussion

Considering the aim of this study we may conclude that cognition is more determined by sociodemographic characteristics rather than ecological assets, while variability in functionality is more linked to ecological assets, namely outdoor mobility and longevity index. As far as depression is concerned, the availability of social resources seems to be the most important ecological asset for ameliorating depression.

With respect to sociodemographic characteristics, there are more women in all age groups, and this number increases as the age group also increases. Illiteracy and lower education are common, mainly, among older elderly, while medium and high education (4–10 years) are typical among younger elderly. The number of widows increases with age, while the number of married elderly decreases. In relation to occupations, most elderly were retired including all elderly aged 85 or older. This way, the sociodemographic profile observed in this sample meets the profile usually described in gerontological literature, a predominance of women, married or widowed persons, retired with no occupation and with low education (Araújo et al., 2007; Espigares et al., 2008; Torres et al., 2010).

The capacity to perform BADL, assessed by Barthel Index, was significantly different among age groups, wherein functional decline was more marked after 85 years of age. This result supports literature that presents a significant increase in dependency for BADL from the fourth age. Santos et al. (2007) found that the incapacity index, measured by Barthel Index, increased from 23,6%, between 60 and 69 years, to 35% between 70 and 79 years, and to 47,7% from 80 years. This steepest functional decline from 85 years old on can be explained, partially, by the fact that most elderly participants of this study are inactive. Furthermore, the literature in the field has shown that the increase of dependency in BADL with age stems from physiological changes and pathologies typical of the aging process (Murtagh and Hubert, 2004; Torres et al., 2010).

In our study, we found significant differences in functionality to IADL, measured by Lawton Index, among age groups. Specifically, there was a more marked decline from 80 years, being more expressive after 85 years old. This data matches those presented in literature which associates the increase in dependency to the increase in age and which shows that the functional decline in IADL is previous to the functional decline in BADL. So, relatively to IADL measured by Lawton Index, Torres et al. (2010) found statistically significant differences according to age group, wherein the elderly with more than 77 years old were more dependent than those with less than 70 years. In the same direction, Costa et al. (2006) and Nakatani et al. (2009) found that, although most elderly participants in their study were independent in BADL, most of them also presented some dependence in IADL. Likewise Nunes et al. (2010) observed that while the functional decline in BADL occurred, mainly, in elderly aged from 80, the functional decline in IADL occurred in elderly aged from 70.

Even in relation to functionality in IADL, the regression analysis showed the 15% of variability is explained by sociodemographic characteristics. Other studies also found that advanced age and lower education are independent factors related to functional dependency (Formiga et al., 2007; Espigares et al., 2008). However, in our study, when the ecological assets were included in the regression analysis, we found that along with sociodemographic characteristics, outdoor mobility explains 32% of the functionality in IADL variability, and when the longevity index is included, this model increases the explanation to 37%. These results suggest that ecological assets should be considered both in the assessment and intervention programs that aim to prevent functional decline.

In what concerns cognition, measured the MMSE, we found statistically significant differences according to age. Specifically, cognitive decline is higher among those participants who are 80 years or older, being more expressive after 85 years. This result fits with several studies that show that there is a general cognitive decline with age, even though age is not the referred as the main factor (Hooren et al., 2007; Bourne et al., 2010; Martínez-Vidal et al., 2011; Oliveira et al., 2011). This cognitive decline can be explained, to a certain point, by the high rate of illiteracy and of lower education in the elderly aged 85 or more, and also by the high number of retired and inactive elderly within this age group. Therefore, the literature has shown, the higher the education level the better the cognitive functioning. This way, Hooren et al. (2007) found that people with higher education had a better cognitive performance, suggesting that this may be due to having greater reserve capacity. Also Bosma et al. (2003) showed that lower education and little cognitively stimulating jobs contributed to a higher cognitive decline. Beyond education and activity, other variables have been associated with cognitive functioning. Specifically, elderly receiving more social support (Bourne et al., 2010), with larger social networks (Holtzman et al., 2004; Green et al., 2008) and married (van Gelder et al., 2006; Karlamangla et al., 2009) had lower cognitive decline. In this study, beyond the 26% of variability in cognitive performance explained by age and education, household and neighborhood indicators, namely outdoor mobility increases the explained variability to 30%, and accessibility indicators provide a further increase to 32%. Again, ecological assets seem to play an important role in cognitive functioning and so they should be included in planned proactive actions for community dwellers.

Concerning depression, there were no significant differences according to age, which is congruent with the literature in the field (Copeland et al., 2004). However, there are higher depression rates from 80 years of age on, which are congruent with the literature showing an increasing trend in depression with age (Beekman et al., 2002; Oliveira et al., 2011). This increase may be related, in this study, to the functional and cognitive decline and to a lesser availability of social resources, diminishing the opportunity for social interaction. Moreover, in the studies of Lenze et al. (2005) and Nunes et al. (2010), depression was a risk factor for incapacity and dependency. Other studies have reported an association with other variables, such as being female (Copeland et al., 2004; McCusker et al., 2005; Tsai et al., 2005; Weele et al., 2009; Oliveira et al., 2011; Seby et al., 2011), low education and socio-economic resources (Tsai et al., 2005; Akyol et al., 2010; Engmann, 2011), living alone (Tsai et al., 2005; Oliveira et al., 2011; Seby et al., 2011), low emotional support (Koizumi et al., 2005), and suffering physical diseases (Oliveira et al., 2011). In the present study, the sociodemographic variables, namely marital status, explain 14% of the variability in depression. However, when the ecological assets are included in the regression analysis, namely social resources the variability explained increases to 18%, suggesting that as the number of leisure opportunities increases depression decreases. These results are relevant because they show that ecological assets, specifically the leisure opportunities, should be integrated in proactive interventions in this field.

Alongside with works of Lerner (Theokas and Lerner, 2006; Urban et al., 2009) regarding the extent of a youth positive development associated with ecological assets, this is the first study showing that besides the acknowledged contribution of sociodemographic variables for aging outcomes, neighborhood ecological assets may have important contributions for ameliorating outcomes such as IADL, cognitive performance and depression in community dwelling old people. Considering the aim of this study we may conclude that cognition has a stronger association with sociodemographic characteristics rather than with ecological assets, while variability in functionality is more linked to ecological assets, namely outdoor mobility and longevity index. As far as depression is concerned, the availability of social resources seems to be the most important ecological asset for ameliorating depression. Aging policies and practices should account for these aspects and be ecologically embedded.

## Conflict of Interest Statement

The Associate Editor Lia Fernandes declares that, despite of having collaborated with the author M. Constança Paul, the review process was handled objectively. The other authors declare that the research was conducted in the absence of any commercial or financial relationships that could be construed as a potential conflict of interest.
